# Prevalence of abnormal thyroid hormone levels in acute new-onset atrial fibrillation

**DOI:** 10.3389/fcvm.2024.1518297

**Published:** 2025-01-10

**Authors:** Jakob Hytting, Serkan Celik, Linus Bodeström Eriksson, Panagiotis Mallios, Christofer Digerfeldt, Annette Waldemar, Magnus Wijkman, Martin Singull, Laila Hubbert

**Affiliations:** ^1^ Department of Cardiology and Department of Health, Medicine and Caring Sciences, Linkoping University, Norrkoping, Sweden; ^2^ Department of Medicine and Department of Health, Medicine and Caring Sciences, Linkoping University, Norrkoping, Sweden; ^3^ Department of Mathematics, Linkoping University, Linkoping, Sweden

**Keywords:** atrial fibrillation, acute new-onset atrial fibrillation, thyroid testing, thyrotoxicosis, subclinical hypothyroidism, hyperthyroidism, emergency department

## Abstract

**Introduction:**

Known risk factors for new-onset atrial fibrillation/flutter (NOAF) include thyrotoxicosis and subclinical hypothyroidism. While prior research has predominantly explored the link between thyrotoxicosis and NOAF, the presence of subclinical hypothyroidism among patients presenting with acute NOAF in the emergency department (ED) remains an underexplored area of inquiry. This study aimed to assess the prevalence of undiagnosed thyrotoxicosis and subclinical hypothyroidism in patients with acute NOAF diagnosed in the ED.

**Methods:**

This registry-based cohort study was conducted in the ED at Vrinnevi Hospital in Sweden during the years 2018, 2020, and 2022, with a 1-year follow-up period. Patients ≥18 years diagnosed with NOAF in the ED, with no ongoing thyroid hormone substitution or previous documented thyroid abnormality within the past 2 years, were included. The primary outcome was the diagnosis of thyrotoxicosis or subclinical hypothyroidism either in the ED or during a 1-year follow-up period.

**Results:**

486 patients with NOAF were included in the study (43.6% females). 329 (67.7%) underwent thyroid function testing in the ED or by the end of the 1-year follow-up. In total, 16 (4.9%) patients presented with subclinical hypothyroidism while 4 (1.2%) patients presented with clinical or subclinical thyrotoxicosis.

**Discussion:**

This study found that subclinical hypothyroidism was more prevalent (4.9%) than thyrotoxicosis (1.2%) among patients presenting with acute NOAF. These findings contrast with previous research that has predominantly linked thyrotoxicosis with acute NOAF, suggesting the need for further studies including both subclinical hypothyroidism and thyrotoxicosis in patients with NOAF.

## Introduction

Atrial fibrillation/flutter (AF) is the most frequent cardiac arrhythmia among patients worldwide (1, 2). The true prevalence of AF is unknown, as many patients remain undiagnosed due to asymptomatic disease (3, 4). AF increases the risk of ischemic stroke, heart failure, sudden cardiac death, and cardiovascular (CV) mortality (1, 2). Various risk factors contribute to the development of AF such as genetic predisposition, advanced age, male sex, overweight, increased alcohol consumption, smoking, CV disease (CVD), and thyroid disease (5, 6). In patients with acute AF, spontaneous conversion is observed in up to 70% of cases. This occurrence depends on factors such as atrial size, concomitant heart failure, previous AF episodes, elevated heart rate, and the presence of a reversible thyrotoxic state (7, 8).

Normal thyroid function is particularly important for maintaining normal cardiac function. Thyroid dysfunctions are common and affects approximately 3.8% of the European population, with an annual incidence of 0.23% for hypothyroidism and 0.05% for hyperthyroidism (9). Clinical thyrotoxicosis, subclinical thyrotoxicosis, and subclinical hypothyroidism are established risk factors for new-onset AF (NOAF) and acknowledged in recent guidelines from the European Society of Cardiology (ESC) (10–13). Accordingly, the ESC recommends thyroid hormone testing in cases of NOAF.

Previous studies have primarily focused on the association between thyrotoxicosis and NOAF, reporting a prevalence ranging from 2.7% to 5.5% in the general NOAF population. In comparison, subclinical hypothyroidism—with an approximate prevalence of 5.7% in the general NOAF population—has received considerably less attention in the literature (14–18). Furthermore, there are few studies evaluating the prevalence of thyroid dysfunctions in patients diagnosed with acute NOAF in the ED. In regard of thyrotoxicosis, one study involving a mixed population of AF patients found that its prevalence was as high as 10.8% (19). However, this study relied solely upon thyroid stimulating hormone (TSH) levels to differentiate thyroid dysfunctions. This method could result in diagnostic issues since TSH profiles can overlap across various thyroid conditions that are not pertinent to AF. In regard of subclinical hypothyroidism, there are to the best of our knowledge, no studies investigating its prevalence in patients with acute NOAF diagnosed in the ED.

The aim of this study was to evaluate the prevalence of clinical and subclinical thyrotoxicosis as well as subclinical hypothyroidism in patients with acute NOAF diagnosed in the ED.

## Materials and methods

### Study design and participants

**Figure 1:**
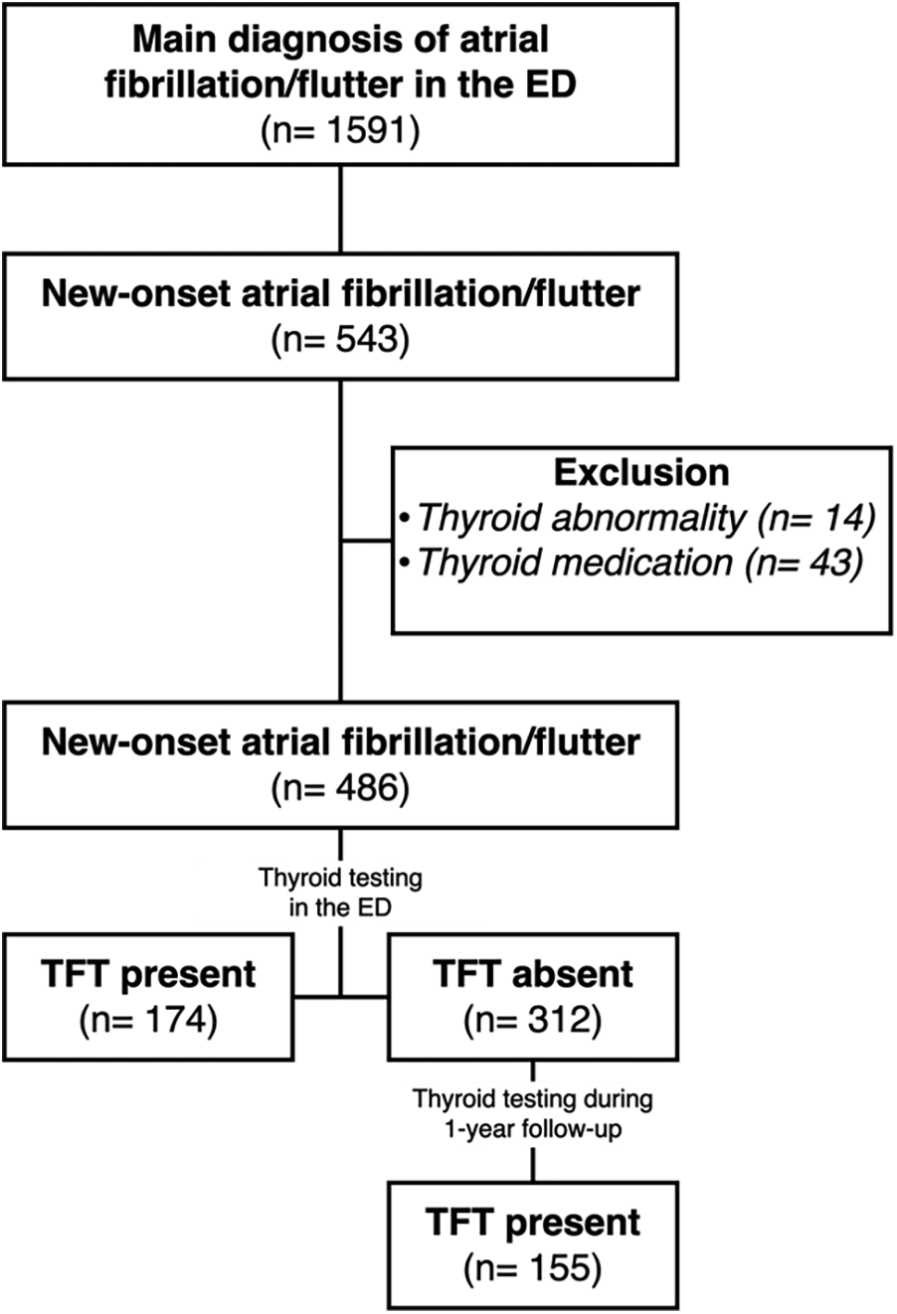
Flow chart depicting exclusion of patients during the eligibility process. ED, emergency department; Prev., previous; TFT, thyroid function testing.

This was a single-center, registry-based, cohort study including all patients aged 18 years or older diagnosed with acute NOAF [International Statistical Classification of Diseases and Related Health Problems (ICD) −10 I48] visiting the ED during the years 2018, 2020, and 2022 at Vrinnevi Hospital, Norrkoping, Sweden (catchment area of 180,000 inhabitants). A review of medical records was performed to confirm acute NOAF diagnosis by interpretation of electrocardiograms (ECGs) (obtained in the ED or in the ambulance). Patients with prior AF, thyroid dysfunction within two years before the ED visit, or those currently prescribed thyroid hormone replacement therapy were excluded. For patients who had multiple emergency department visits during the study period, data from their initial admission was included, while information from any subsequent re-admissions was excluded, (Figure 1).

The study was approved by the Swedish Ethics Review Authority (2021-05963-01) and adhered to the principles of the Declaration of Helsinki. According to Swedish legislation, patients registered in healthcare quality registers are not required to provide written informed consent for their data to be used in healthcare research or publication (20).

### Data sources

Data were obtained by Region Ostergotland decision support and follow-up system (REBUS) and medical records using the unique national identification number assigned to every Swedish resident at birth or at residency. Personal identification numbers given at birth or residency indicate the assigned sex by the penultimate number. Data collected from REBUS included date of visit at the ED, diagnosis (ICD 10), age, sex, date of discharge, and date of death. Data at the time of AF diagnosis regarding: CV risk factors (CVRF) [smoking, obesity (body mass index BMI ≥30 kg/m^2^)] and comorbidities [heart failure (ICD-10 I50), ischemic heart disease (I20-I25), diabetes mellitus (E10), hypertension (I10)] were recorded. Additionally, heart rhythm (from ECG) and lab results [high sensitivity Troponin T (hsTnT), N-Terminal pro-B-type Natriuretic Peptide (NT-proBNP), hemoglobin, C-Reactive Protein (CRP), and blood glucose] were extracted from the medical records.

### Thyroid testing and interpretation

TSH and free thyroxine (fT4) levels were obtained from medical records at the ED visit, as well as those lab results recorded within two years prior to the ED visit. Since the regional guidelines for thyrotoxicosis recommend initial testing with only TSH and fT4 in patients with a suspicion of hyperthyroidism, we did not have information regarding triiodothyronine (fT3) levels. Furthermore, for patients who had not undergone a thyroid function test either previously or in the emergency department, TSH and fT4 levels were evaluated during a 1-year follow-up period through their medical records.

Thyroid dysfunctions were classified according to local reference values (TSH 0.3–4.2 mIE/L and fT4 12–22 pmol/L), (Supplementary Table S1) (21–23). Thyroid function tests were analyzed with electrochemiluminescence immunoassay using Cobas e601 and e602 (Roche, Basel, Switzerland).

### Outcome

The primary outcome was the detection of clinical or subclinical thyrotoxicosis as well as subclinical hypothyroidism in patients diagnosed with NOAF in the ED.

### Statistical analysis

Baseline data is presented for the entire cohort and then divided into two groups: patients who received thyroid tests and patients who did not. The Kolmogorov-Smirnov test was used on continuous variables to determine whether data followed normal distribution. Continuous data, where normal distribution was not rejected, were presented with mean and standard deviation (SD). Continuous data with skewed distribution were presented with median with interquartile range (IQR). Categorical data were presented as number (*n*) and percent (%). Between-group differences regarding qualitative data were tested for statistical significance using the chi-squared test. If more than 20% of cells had a frequency <5 Fisher's exact test was used. The two sample *t*-test was used for comparison of normally distributed variables between two groups of independent samples, and the Mann-Whitney *U*-test was used on continuous variables that had a skewed distribution.

For statistical analysis, IBM SPSS, Statistics, 28.0 (Armonk, NY, USA) was used. A value of *p* < 0.05 was set as level of statistical significance.

## Results

The study included 1,591 patients with AF during the years of 2018, 2020, and 2022. Of these, 486 patients [median age 73.1 years (IQR 62.6–79.8) and 43.6% female] were eligible for analysis, (Figure 1). Comorbidities were common, including hypertension (63.2%), obesity (34.8%), heart failure (16.9%), ischemic heart disease (18.9%), and type 2 diabetes mellitus (14.7%). Baseline characteristics are presented in Table 1.

**Table 1:** Demographic and clinical characteristics of 486 patients with new-onset atrial fibrillation/flutter in the emergency department at Vrinnevi hospital Norrkoping, Sweden in 2018, 2020, and 2022.

*n* (% of total)	Total 486	Thyroid testing in the ED 174 (35.8)	No thyroid testing in the ED 312 (64.2)	*p*-value
Female, *n* (%)	212 (43.6)	76 (43.6)	136 (43.6)	0.985
Median age, years median (IQR)	73.1 (62.6–79.8)	69.3 (56.4–76.8)	75.0 (66.5–80.9)	<0.001
Age groups, *n* (%)				<0.001
<60 years	98 (20.2)	54 (31.0)	44 (14.1)	
61–70 years	98 (20.2)	36 (20.7)	62 (19.9)	
71–80 years	175 (36.0)	57 (32.8)	118 (37.8)	
≥80 years	115 (23.7)	27 (15.5)	88 (28.2)	
Comorbidities and CVRF, *n* (%)				
Heart failure	82 (16.9)	25 (14.4)	57 (18.3)	0.271
Ischemic heart disease	91 (18.7)	32 (18.4)	59 (18.9)	0.888
Diabetes mellitus type 2	72 (14.8)	19 (10.9)	53 (17.0)	0.071
Hypertension	307 (63.2)	98 (56.3)	209 (67.0)	0.019
Smoking^a^	61 (13.9)	23 (14.2)	38 (13.7)	0.889
Obesity (BMI >30 kg/m^2^)^b^	146 (34.8)	59 (38.8)	87 (32.6)	0.198
1-year mortality, *n* (%)	42 (8.6)	14 (8.0)	28 (9.0)	0.727
Heart rate, beats/min mean (SD)	124 (30)	127 (29)	122 (31)	0.064
ECG rhythm, *n* (%)				
Atrial fibrillation	400 (82.3)	138 (79.3)	262 (84.0)	0.245
Atrial flutter	84 (17.3)	36 (20.7)	48 (15.4)	
Pacemaker rhythm	2 (0.4)	0 (0)	2 (0.6)	
Laboratory findings				
hsTnT, ng/L median (IQR)^c^	17 (9–28)	16 (9–25)	18 (9–28)	0.257
NT-pro-BNP, ng/L median (IQR)^d^	1,825 (658–4,143)	1,410 (485–3,982)	2,030 (840–4,235)	0.168
TSH, mIE/L median (IQR)	–	1.7 (1.2–2.9)	–	na
fT4, pmol/L median (IQR)	–	16.4 (15.0–18.1)	–	na
Hemoglobin, g/L median (IQR)^e^	144 (133–154)	146 (136–154)	143 (132–154)	0.078
CRP, mg/L median (IQR)^f^	2.5 (2.5–10)*	2.5 (2.5–14)*	2.5 (2.5–8)*	0.023
Creatinine, µmol/L median (IQR)^e^	81 (69–97)	79 (67–95)	83 (71–99)	0.056
Blood glucose, mmol/L median (IQR)^g^	6.3 (5.4–7.0)	6.3 (5.5–6.9)	6.1 (5.3–6.8)	0.928
Previous thyroid testing, *n* (%)	129 (26.5)	31 (17.8)	98 (31.4)	0.001

*P*-values are presented for the groups: thyroid testing in the ED vs. no thyroid testing in the ED. ED, emergency department; ECG, electrocardiography; CVRF, cardiovascular risk factors; BMI, body mass index; hsTnT, high sensitivity Troponin-T; NT-pro-BNP, N-terminal pro-B-type natriuretic peptide; TSH, thyroid stimulating hormone; fT4, free thyroxine hormone; CRP, C-reactive protein; n, numbers; SD, standard deviation; IQR, interquartile range; na, not applicable.

Data missing for:

^a^ 47 patients.

^b^ 67 patients.

^c^ 72 patients.

^d^ 330 patients.

^e^ 3 patients.

^f^ 43 patients.

^g^ 266 patients.

* For analytical purposes, CRP levels below the lower level of detection (i.e., values <5) were assigned the value of 2.5.

Out of the 486 patients diagnosed with acute NOAF, 174 (35.8%) underwent thyroid function testing in the ED (43.6% females). During the 1-year follow-up, an additional 155 patients were tested (49.0% females), bringing the total to 329 (67.7%) patients who underwent thyroid testing within the first year of their NOAF diagnosis (46.2% females), (Figure 1). For all the tested patients, the median TSH level was 1.7 (1.2–2.9) mIE/L and fT4 level was 16.4 (15.0–18.1) pmol/L.

**Figure 2:**
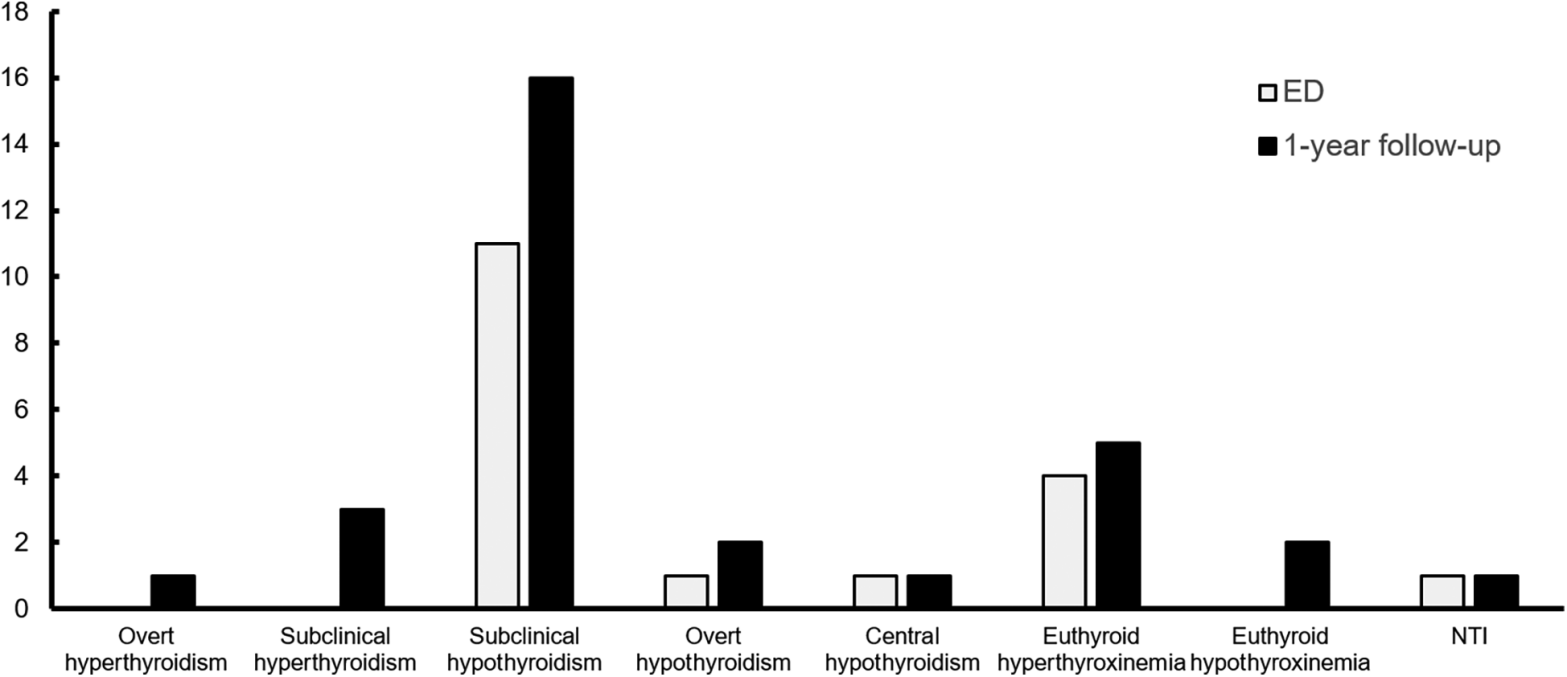
Distribution of thyroid dysfunction in the ED and by the end of the 1-year follow-up. ED, emergency department; NTI, non-thyroid illness.

Overall, 31 patients (9.4%) with NOAF were found to have previously undiagnosed thyroid hormone abnormalities. Among them, 16 patients (4.9%) had subclinical hypothyroidism, and 4 patients (1.2%) had clinical or subclinical thyrotoxicosis. Regarding subclinical hypothyroidism, 11 patients (3.3%) were diagnosed in the ED, while an additional 5 patients (1.6%) were diagnosed during the 1-year follow-up. Regarding thyrotoxicosis, no cases of clinical or subclinical thyrotoxicosis were diagnosed in the ED, while 1 patient (0.3%) was diagnosed with clinical thyrotoxicosis and 3 patients (0.9%) were diagnosed with subclinical thyrotoxicosis during the 1-year follow-up, (Figure 2). No patients with confirmed thyroid dysfunction received Amiodarone prior to their thyroid function testing, 1 patient diagnosed with subclinical hypothyroidism later received Amiodarone in an outpatient setting. Additionally, 4 patients diagnosed with subclinical hypothyroidism and 1 patient with overt thyrotoxicosis later presented with normalized thyroid function within the follow-up period (re-evaluation occurred within 1–5 months after the initial testing for all but one patient, where it occurred within 9 months). Furthermore, 8 patients diagnosed with subclinical hypothyroidism and 2 patients with subclinical thyrotoxicosis had no re-evaluation within the 1-year follow-up, (Supplementary Table S2).

Patients screened for thyroid dysfunction in the ED were significantly younger (median age 69.3 vs. 75.0 years) than those who were not tested (*p* < 0.001), had a lower incidence of hypertension (56.3% vs. 67.0%, *p* = 0.019), and were less likely to have undergone previous thyroid testing (17.8% vs. 31.4%, *p* = 0.001) compared to those not tested. Differences were also observed in CRP levels between the groups (*p* = 0.023), although the median CRP level for both groups was the same at 2.5 mg/L, (Table 1). Additionally, patients tested and then diagnosed with clinical thyrotoxicosis, subclinical thyrotoxicosis, or subclinical hypothyroidism did not significantly differ in clinical characteristics when compared to those with normal thyroid function, (Table 2).

**Table 2:** Demographic and clinical characteristics of patients with normal thyroid function and thyroid dysfunctions associated with new-onset atrial fibrillation/flutter diagnosed in the emergency department at Vrinnevi hospital Norrkoping, Sweden in 2018, 2020, and 2022.

*n* (% of total)	Normal thyroid function 298 (93.7)	Thyroid dysfunctions* 20 (6.3)	*p*-value
Female, *n* (%)	133 (44.6)	12 (60.0)	0.182
Median age, years (IQR)	72.0 (61.0–78.0)	74.5 (58.25–81.0)	0.468
Comorbidities and CVRF, *n* (%)			
Heart failure	51 (17.1)	4 (20.0)	0.741
Ischemic heart disease	55 (18.5)	3 (15.0)	0.673
Diabetes mellitus type 2	36 (12.1)	1 (5.0)	0.488
Hypertension	191 (64.1)	15 (75.0)	0.323
Smoking^a^	38 (12.8)	3 (18.8)	0.573
Obesity (BMI >30 kg/m^2^)^b^	93 (31.2)	7 (41.2)	0.628
1-year mortality, *n* (%)	19 (6.4)	1 (5.0)	1.000
Heart rate, beats/min mean (SD)	125 (30)	127 (34)	0.790
ECG rhythm, *n* (%)			
Atrial fibrillation	242 (81.2)	16 (80.0)	1.000
Atrial flutter	56 (18.8)	4 (20.0)	
Pacemaker rhythm	0 (0)	0 (0)	
Laboratory findings			
hsTnT, ng/L median (IQR)^c^	16 (10–25)	24 (16–40)	0.037
NT-pro-BNP, ng/L median (IQR)^d^	1,730 (620–3,828)	4,520 (390–6,070)	0.309
Hemoglobin, g/L median (IQR)^e^	144 (134–153)	145 (131–153)	0.974
CRP, mg/L median (IQR)^f^	2.5 (2.5–9.0)**	6.0 (2.5–32)**	0.200
Creatinine, µmol/L median (IQR)^e^	81 (68–95)	77 (67–93)	0.558
Blood glucose, mmol/L median (IQR)^g^	6.1 (5.4–6.9)	7.0 (5.6–7.4)	0.287
Previous thyroid testing, *n* (%)	76 (25.5)	5 (25.0)	0.960

ED, emergency department; ECG, electrocardiography; CVRF, cardiovascular risk factors; BMI, body mass index; hsTnT, high sensitivity Troponin-T; NT-pro-BNP, N-terminal pro-B-type natriuretic peptide; TSH, thyroid stimulating hormone; fT4, free thyroxine hormone; CRP, C-reactive protein; n, numbers; SD, standard deviation; IQR, interquartile range; na, not applicable.

Data missing for:

^a^ 21 patients.

^b^ 35 patients.

^c^ 44 patients.

^d^ 194 patients.

^e^ 1 patient.

^f^ 27 patients.

^g^ 163 patients.

* Clinical thyrotoxicosis, subclinical thyrotoxicosis, and subclinical hypothyroidism.

** For analytical purposes, CRP levels below the lower level of detection (i.e., values <5 mg/L) were assigned the value of 2.5.

## Discussion

Abnormal thyroid hormone levels are an established risk factor for NOAF and recently subclinical hypothyroidism has been highlighted as a clinically relevant disturbance in patients with NOAF (13, 24). The findings in the current study are, to the best of our knowledge, the first to show that subclinical hypothyroidism might be more prevalent than thyrotoxicosis in patients with acute NOAF diagnosed in the ED, with a prevalence of 4.9% compared to 1.2%.

Thyroid dysfunctions are sparsely studied in acute NOAF diagnosed in the ED, and studies have primarily focused on thyrotoxicosis. Buccelletti et al. (19) found by analyzing TSH levels solely that thyrotoxicosis was common, suggesting a prevalence as high as 10.8% vs. 1.2% in the current study. This discrepancy may be attributed to various factors, such as differences in diagnostic thresholds and laboratory assays when diagnosing thyrotoxicosis as well as differences in populations and geographic variations in thyroid disease prevalence. It should be noted that there are differences in the annual incidence of hyperthyroidism in Sweden compared to i.e., northern Italy (0.03% vs. 0.08% respectively), which may account for some of the observed difference (25, 26). Furthermore, when differentiating thyroid disorders, it is essential to understand that TSH levels alone are insufficient for distinguishing between clinical thyrotoxicosis, subclinical thyrotoxicosis, non-thyroidal illness, and central hypothyroidism, as well as between clinical and subclinical hypothyroidism. To improve accuracy and decrease the risk of overestimating non-significant thyroid dysfunctions, additional factors like the clinical context and biomarkers such as fT4 should be included (23).

Conversely, the prevalence of thyroid dysfunctions in the general NOAF population has been extensively studied, but the focus has primarily been on thyrotoxicosis, while subclinical hypothyroidism has often been overlooked. However, a study by Selmer et al. (12) employed a comprehensive diagnostic approach that included both TSH and fT4 levels and presented data on thyrotoxicosis as well as subclinical hypothyroidism, an approach adopted in the current study. Selmer et al. reported a prevalence of 2.7% for thyrotoxicosis and 5.7% for subclinical hypothyroidism, findings that align with the current study. Furthermore, Boriani et al. showed a similar prevalence of thyrotoxicosis (3%) in the general NOAF population. Additionally, Boriani et al. (16) included cases of hypothyroidism but did not differentiate between subclinical and clinical cases. Krahn et al. (15) reported a higher prevalence of thyrotoxicosis (5.4%) in the general NOAF population; however, they did not account for subclinical hypothyroidism and relied on TSH levels for diagnosis. As previously noted, this approach may lead to an overestimation of thyrotoxic cases. The above findings, in synthesis with the findings in the current study, suggest that the prevalence of significant thyroid dysfunctions in acute NOAF diagnosed in the ED is similar to that observed in the general NOAF population.

This study used data from high-quality patient registers with low dropout rates. Nevertheless, studies based on registers and patient medical records invariably encounter issues such as selection bias, confounding variables, measurement inaccuracies, and reporting biases. The hospital houses the only ED in the catchment area, providing a comprehensive real-life population of all NOAF patients diagnosed in the ED within the region. However, even though this study covers the total population of NOAF in the catchment area, the cohort size is limited. While the cohort size is similar to Buccelletti et al. (19), it is smaller than in recent studies of the general NOAF population (16, 17, 19). Within this cohort, only a third underwent thyroid testing in the ED and two thirds totally during the 1-year follow-up which may limit the generalizability of the results. Furthermore, it should be acknowledged that those with newly detected thyroid abnormalities during follow-up may not have had a thyroid dysfunction at the time of admission to the ED, and previous studies have found that NOAF is an independent predictor of subsequent thyrotoxicosis (17, 27). The 1-year follow-up in this study introduces a potential risk of overestimating the prevalence of thyrotoxicosis in the ED; nonetheless, the observed prevalence was notably lower than previously reported. Given that NOAF serves as a risk factor for subsequent thyrotoxicosis and considering the lower prevalence in this study compared to previous studies, the 1-year follow-up might not introduce a significant weaknesses in the study design. Subclinical hypothyroidism has been shown to resolve spontaneously in a majority of cases, suggesting that the follow-up could lead to an underestimation of its prevalence (28). The prevalence of subclinical hypothyroidism tends to increase with age, and the median age of 73 years in the current study may have contributed to the higher prevalence observed compared to thyrotoxicosis. This age-related trend highlights the importance of considering demographic factors when interpreting differences in prevalence between these conditions (29). Additionally, the absence of fT3 levels in the current study is a limitation. However, it does not significantly impact the findings, as the comprehensive use of TSH and fT4 levels still provides a robust assessment of thyroid function.

The clinical implications of these findings are yet to be determined, and the effects of treating subclinical hypothyroidism in patients with NOAF needs further inquiry (24, 30). As recently reviewed, most guidelines recommend treatment for subclinical hypothyroidism only for patients exhibiting symptoms of hypothyroidism, or for those with TSH levels exceeding 10 mIE/L (31). Observational data have suggested a potential CV benefit from active treatment but randomized trials are needed for any firm conclusions to be drawn (32). Whether NOAF should be considered a symptom of subclinical hypothyroidism remains uncertain but cases of AF resolution have been documented following the treatment of subclinical hypothyroidism (33, 34). Larger multicenter studies involving diverse populations seem to be needed to confirm the prevalence rates of subclinical hypothyroidism and thyrotoxicosis in acute NOAF patients. Exploring geographic and demographic variations could identify populations at higher risk and inform targeted interventions to increase adherence to ESC guidelines regarding thyroid hormone testing. Furthermore, younger age and the absence of previous thyroid function testing were factors associated with a higher frequency of thyroid testing in NOAF. However, these factors did not significantly differentiate between those with a diagnosed thyroid disease and those without. This suggests that while certain demographic and clinical factors may influence the decision to conduct thyroid testing, they are not reliable indicators of underlying thyroid pathology in acute NOAF.

## Conclusion

In conclusion, subclinical hypothyroidism was more frequently prevalent than thyrotoxicosis (4.9% vs. 1.2%) in patients diagnosed with acute NOAF, suggesting it may play a more significant role than previously recognized. These findings suggest that there is a need for further studies including both thyrotoxicosis as well as subclinical hypothyroidism in acute NOAF and further research into the clinical implications of subclinical hypothyroidism in this population.

## Data Availability

The raw data supporting the conclusions of this article will be made available by the authors, without undue reservation.
