# Supplementary material for: Prevalence of abnormal thyroid hormone levels in acute new-onset atrial fibrillation
Source: Front Cardiovasc Med. 2025 Jan 10;11:1518297. doi: 10.3389/fcvm.2024.1518297 (PMC11757249; doi:10.3389/fcvm.2024.1518297)
Supplement: Supplementary file 1 [file Table1.docx]

| **Supplementary Table 1.** Classification of thyroid dysfunctions | | | |
| --- | --- | --- | --- |
| **Pattern of dysfunction** | **TSH**  **(ref 0.3-4.2 mIE/L)** | **fT4**  **(ref 12-22 pmol/L)** | **Comments** |
| Overt hypothyroidism | >4.2 mIE/L | <12 pmol/L |  |
| Subclinical hypothyroidism | >4.2 mIE/L | 12-22 pmol/L |  |
| Overt thyrotoxicosis | <0.3 mIE/L | >22 pmol/L |  |
| Subclinical thyrotoxicosis | <0.3 mIE/L | 12-22 pmol/L |  |
| Non-thyroid illness* | Variable | Variable | Concomitant severe illness |
| Euthyroid hyperthyroxinemia | Normal | >22 pmol/L |  |
| Euthyroid hypothyroxinemia | Normal | <12 pmol/L |  |
| Central hypothyroidism* | ≤4.2 mIE/L | <12 pmol/L |  |
| TSH, Thyroid stimulating hormone; fT4, free thyroxine hormone.  * Patients with either non-thyroid illness (n=1) or central hypothyroidism (n=1) were classified according to thorough review of medical records. | | | |
