# Supplementary material for: Prevalence of abnormal thyroid hormone levels in acute new-onset atrial fibrillation
Source: Front Cardiovasc Med. 2025 Jan 10;11:1518297. doi: 10.3389/fcvm.2024.1518297 (PMC11757249; doi:10.3389/fcvm.2024.1518297)
Supplement: Supplementary file 2 [file Table2.docx]

| **Supplementary table 2.** Characteristics of thyroid dysfunctions* in the ED or during the 1-year follow-up | | | | | |
| --- | --- | --- | --- | --- | --- |
|  | **Type of thyroid dysfunction** | **TSH** | **fT4** | **Additional information** | **Re-evaluation**** |
| 1 | Subclinical hypothyroidism | 4.4 | 16.2 | TFT in the ED  Later received Amiodarone | TF normalized |
| 2 | Subclinical hypothyroidism | 6.5 | 18.7 | TFT in the ED | TF normalized |
| 3 | Subclinical hypothyroidism | 5.2 | 16.4 | TFT in the ED | TF normalized |
| 4 | Subclinical hypothyroidism | 4.7 | 14.,9 | TFT in the ED | TF normalized |
| 5 | Subclinical hypothyroidism | 4.5 | / | TFT in the ED | TF normalized |
| 6 | Subclinical hypothyroidism | 4.8 | 12.6 | TFT in the ED | Pathology remained |
| 7 | Subclinical hypothyroidism | 9.7 | 13.4 | TFT in the ED | Pathology remained |
| 8 | Subclinical hypothyroidism | 5.4 | 16.6 | TFT in the ED | Pathology remained |
| 9 | Subclinical hypothyroidism | 4.4 | 17.3 | TFT in the ED  Later received THS | Pathology remained |
| 10 | Subclinical hypothyroidism | 4.8 | 15.2 | TFT in the ED | No reevaluation available |
| 11 | Subclinical hypothyroidism | 5.0 | 14.8 | TFT in the ED | No reevaluation available |
| 12 | Subclinical hypothyroidism | 5.9 | 17.5 | TFT 7 days after ED-visit | No reevaluation available |
| 13 | Subclinical hypothyroidism | 6.0 | 15.6 | TFT 12 days after ED-visit | No reevaluation available |
| 14 | Subclinical hypothyroidism | 4.6 | 17.0 | TFT 36 days after ED-visit | No reevaluation available |
| 15 | Subclinical hypothyroidism | 5.7 | / | TFT 73 days after ED-visit | No reevaluation available |
| 16 | Subclinical hypothyroidism | 6.5 | 15.0 | TFT 87 days after ED-visit | No reevaluation available |
| 17 | Subclinical thyrotoxicosis | 0.21 | 17.1 | TFT 69 days after ED-visit | Pathology remained |
| 18 | Subclinical thyrotoxicosis | 0.20 | 18.3 | TFT 80 days after ED-visit | No reevaluation available |
| 19 | Subclinical thyrotoxicosis | 0.24 | 18.6 | TFT 262 days after ED-visit | No reevaluation available |
| 20 | Clinical thyrotoxicosis | 0.23 | 29.3 | TFT 40 days after ED-visit | TF normalized |
| TSH, Thyroid stimulating hormone; fT4, free thyroxine hormone; ED, emergency department; TF, thyroid function, TFT, thyroid function testing; THS, thyroid hormone substitution.  *Clinical thyrotoxicosis, subclinical thyrotoxicosis, and subclinical hypothyroidism  **Re-evaluation occurring within the timeframe of the 1-year follow-up | | | | | |
